# Vitamin K-Dependent Protein Activation: Normal Gamma-Glutamyl Carboxylation and Disruption in Disease

**DOI:** 10.3390/ijms23105759

**Published:** 2022-05-20

**Authors:** Kathleen L. Berkner, Kurt W. Runge

**Affiliations:** 1 Department of Cardiovascular and Metabolic Sciences, Lerner Research Institute, Cleveland Clinic Lerner College of Medicine at CWRU, Cleveland, OH 44195, USA; 2 Department of Inflammation and Immunity, Lerner Research Institute, Cleveland Clinic Lerner College of Medicine at CWRU, Cleveland, OH 44195, USA; rungek@ccf.org

**Keywords:** processivity, vitamin K, vitamin K-dependent proteins, gamma-glutamyl carboxylase (GGCX), vitamin K oxidoreductase (VKORC1), warfarin, VKCFD, pseudoxanthoma elasticum-like (PXE-like)

## Abstract

Vitamin K-dependent (VKD) proteins undergo an unusual post-translational modification, which is the conversion of specific Glu residues to carboxylated Glu (Gla). Gla generation is required for the activation of VKD proteins, and occurs in the endoplasmic reticulum during their secretion to either the cell surface or from the cell. The gamma-glutamyl carboxylase produces Gla using reduced vitamin K, which becomes oxygenated to vitamin K epoxide. Reduced vitamin K is then regenerated by a vitamin K oxidoreductase (VKORC1), and this interconversion of oxygenated and reduced vitamin K is referred to as the vitamin K cycle. Many of the VKD proteins support hemostasis, which is suppressed during therapy with warfarin that inhibits VKORC1 activity. VKD proteins also impact a broad range of physiologies beyond hemostasis, which includes regulation of calcification, apoptosis, complement, growth control, signal transduction and angiogenesis. The review covers the roles of VKD proteins, how they become activated, and how disruption of carboxylation can lead to disease. VKD proteins contain clusters of Gla residues that form a calcium-binding module important for activity, and carboxylase processivity allows the generation of multiple Glas. The review discusses how impaired carboxylase processivity results in the pseudoxanthoma elasticum-like disease.

## 1. Introduction

Dietary vitamin K is used in virtually all tissues to activate vitamin K-dependent (VKD) proteins through the conversion of specific glutamyl (Glu) residues to carboxylated Glu (Gla) [1,2]. VKD proteins have broad biological impact, as they are important in diverse physiologies that include hemostasis, regulation of calcification, inflammation, angiogenesis, growth control, complement and apoptosis [3]. Disruption of VKD protein carboxylation leads to multiple pathologies, including bleeding and aberrant calcification. VKD proteins are carboxylated in the endoplasmic reticulum during their secretion, and carboxylation involves several proteins that participate in a complex process [1,2]. Two of these proteins, the gamma-glutamyl carboxylase and vitamin K oxidoreductase C1 (VKORC1), modify VKD proteins through a vitamin K cycle: the carboxylase generates Gla residues by using the oxygenation of reduced vitamin K, and VKORC1 regenerates reduced vitamin K to support carboxylase activity. VKORC1 is the target of anticoagulants like warfarin (Coumadin), which is used by millions of people to prevent thrombotic events. Warfarin therapy is associated with both vascular and cardiac valvular calcification [4,5,6,7,8]. Dysregulation of calcification also occurs in the pseudoxanthoma elasticum (PXE)-like disease, which is caused by some mutations in the carboxylase and is associated with aberrant calcification in skin as well as mildly defective bleeding [9,10,11,12,13]. Other carboxylase mutations, as well as a VKORC1 mutation, cause VKCFD (for vitamin K clotting factor deficiency) 1 and 2, respectively [14]. This review covers the roles of VKD proteins, how they become activated, and how this process is disrupted in disease. It also emphasizes novel insights into the essentiality of carboxylase processivity, i.e., in which the carboxylase remains bound to VKD proteins to generate multiple Gla residues. The cluster of Gla residues forms a calcium-binding module in VKD proteins required for activity, and recent studies [15,16] show that disrupted processivity results in the PXE-like disease.

## 2. Vitamin K-Dependent Proteins Are Essential to Human Health

**Figure 1:**
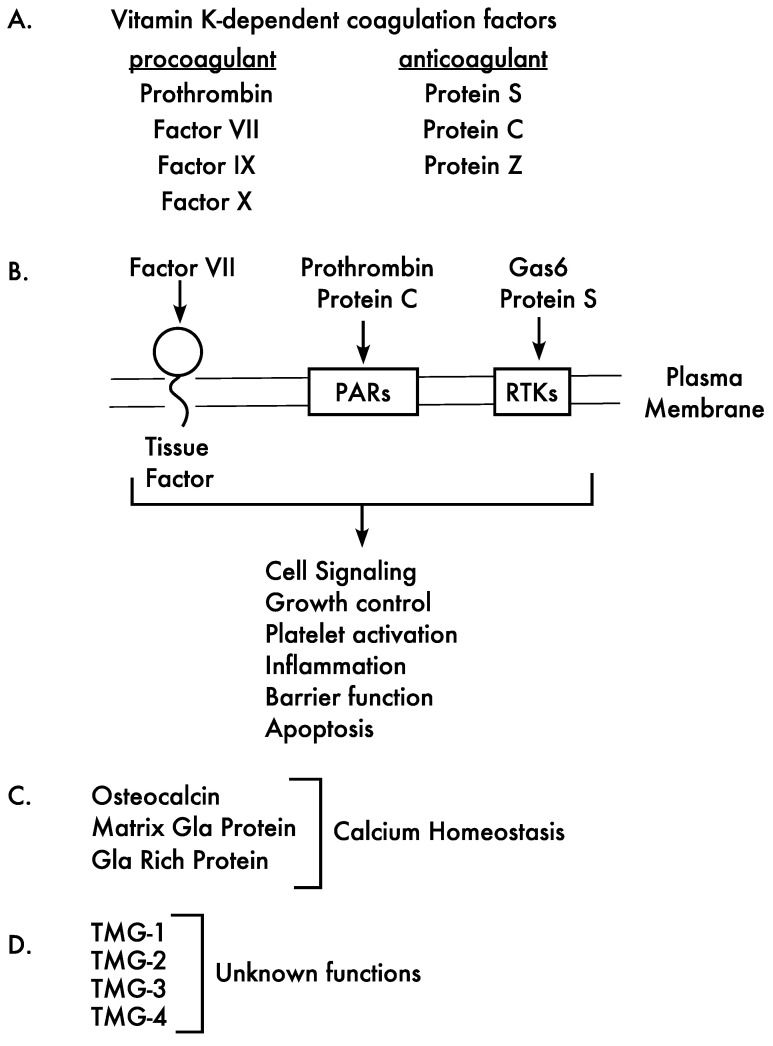
Vitamin K-dependent proteins impact multiple physiologies. (**A**). Many of the vitamin K-dependent proteins are essential for blood clotting, either supporting (procoagulant) or attenuating (anticoagulant) hemostasis. (**B**). Msost of the coagulation factors, as well as Gas6, signal through various receptors on the cell surface. Some examples of receptors are tissue factor, platelet activated receptors (PARS) and receptor tyrosine kinases (RTKs), which mediate multiple physiologies. The scheme shows zymogen nomenclatures for simplicity; however, the function of VKD coagulation factors requires activation (e.g., of prothrombin to thrombin). (**C**,**D**). VKD proteins that have roles beyond hemostasis have also been identified. Some regulate calcification (**C**), while others have functions that remain to be determined (**D**). TMG stands for Transmembrane Gla Protein, a family of proline-rich proteins that are also referred to as PRGP.

Vitamin K was originally implicated as important to hemostasis by the hemorrhagic response of chicks fed a lipid free diet. Subsequent discoveries that were significant in understanding the role of vitamin K were its isolation and identification, the identification of VKD proteins, and the demonstration that these proteins undergo an unusual post-translational modification, which is the carboxylation of specific Glu residues to Glas [1,2,3]. The first VKD proteins identified were hemostatic VKD proteins, which have been extensively studied, including efforts that unequivocally showed that carboxylation is required for activity. Liver is the major site of synthesis of the hemostatic VKD proteins, which are secreted into blood and respond to injury through the coagulation cascade [17]. This pathway involves a series of proteolytic cleavage reactions, which ultimately results in the formation of a fibrin clot that seals the site of injury to stem blood loss. Some of the VKD clotting factors are procoagulant, while others have anticoagulant roles that suppress the coagulation cascade once the clot has formed (Figure 1A). Many of the coagulant proteins also have signaling roles that can impact hemostasis (Figure 1B), for example thrombin (the active form of prothrombin) activation of platelets through platelet activated receptors [18]. Platelet activation is essential for wound repair because it results in the incorporation of platelets into the fibrin clot, and platelet activation is also mediated by the VKD protein Gas6 [19,20], which is not a coagulation protein. Many of the hemostatic VKD proteins are also expressed in extrahepatic tissues, where they have additional roles, for example protein S that impacts complement and phagocytosis, protein C that regulates inflammation and barrier function, and Gas6 that functions in growth control [19,20,21,22,23,24,25,26] (Figure 1B).

An even broader impact of vitamin K on human health has been revealed by the identification of nonhemostatic VKD proteins (Matrix Gla Protein (MGP), osteocalcin, Gla Rich Protein (GRP) and four transmembrane proteins (TMGs) (Figure 1C,D). MGP is broadly expressed, including in the vasculature where it has been shown to be a potent inhibitor of calcification by the phenotype of *mgp^−/−^* mice, who die shortly after birth due to massive arterial calcification and vascular rupture [27]. Warfarin, which is a vitamin K antagonist, causes rapid arterial and aortic valve calcification in rodents that is associated with decreased MGP carboxylation [28,29]. In humans, MGP mutations cause Keutel syndrome that is characterized by abnormal cartilage calcification [30]. Mutations in the carboxylase that modifies the VKD proteins also result in aberrant calcification, as described in more detail below. GRP is also a calcification inhibitor in the cardiovascular system [31,32,33,34,35], although the *grp^−/−^* mice do not reveal a clear phenotype as in the case of the *mgp^−/−^* mice. The remaining nonhemostatic proteins, i.e., the TMG proteins, are distinct from all other known VKD proteins in having a single pass transmembrane sequence that localizes the Gla domain at the exterior of the plasma membrane. These proteins are expressed in a wide variety of tissues and have motifs suggesting roles in signal transduction [36,37]; however their functions have not been determined.

## 3. Multiple Proteins Cooperate in Activating VKD Proteins during Secretion

### 3.1. Carboxylation, Secretion and Quality Control

**Figure 2:**
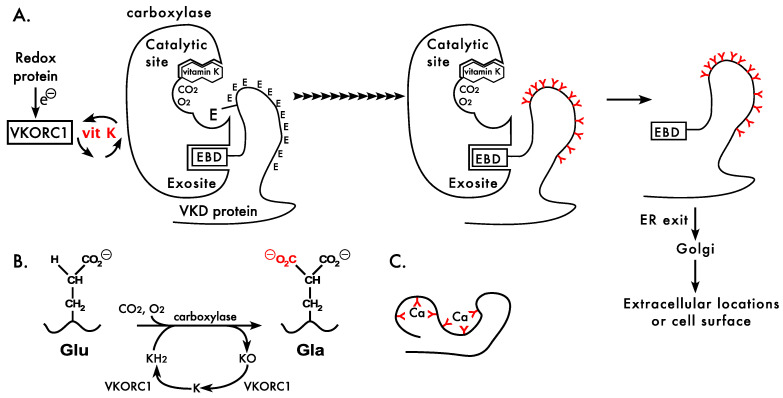
Carboxylation of multiple glutamyl residues generates a calcium-binding module required for vitamin K-dependent protein activities. (**A**) Vitamin K-dependent (VKD) protein carboxylation occurs in the endoplasmic reticulum, where vitamin K (vit K) cycles between reduced vitamin K generated by a vitamin K epoxide reductase (VKORC1) and oxidized vitamin K produced by the carboxylase. VKORC1 becomes inactivated during vitamin K reduction, and activity is regenerated through electron (e^−^) flow from a redox protein. VKD proteins are targeted for carboxylation because they contain an exosite binding domain (EBD) that mediates binding to the carboxylase. Multiple Glu residues are carboxylated (arrowheads) to Glas (red Ys). Following carboxylation and release from the carboxylase, the VKD proteins exit the endoplasmic reticulum (ER) and traffic to the Golgi where additional post-translational modifications occur. Further secretion localizes VKD proteins in blood or extracellular matrix or the cell surface. (**B**) The carboxylase uses the epoxidation of vitamin K hydroquinone (KH_2_) to vitamin K epoxide (KO) to replace a hydrogen in glutamic acid (Glu) residues by CO_2_, forming carboxylated Glu (Gla). KO is then recycled by VKORC1, first to a quinone intermediate (K) and then to vitamin K hydroquinone (KH_2_). (**C**) Multiple Gla residues (red Ys) coordinate several calcium ions to transform the Gla domain into a highly organized structure.

VKD proteins undergo the conversion of Glus to Glas in the endoplasmic reticulum during secretion [1] (Figure 2). These proteins are selectively modified because they contain a specific sequence, i.e., an exosite binding domain (EBD), that mediates binding to the gamma-glutamyl carboxylase (Figure 2A). In most cases, the EBD in VKD proteins is a propeptide that is cleaved after carboxylation. MGP has a distinct structure, as the EBD sequence is internal and retained within the mature protein. Following carboxylation, the VKD proteins exit the endoplasmic reticulum and traffic to the Golgi where additional post-translational modifications occur. Factor IX, for example, undergoes N- and O-glycosylation, tyrosine sulfation, serine phosphorylation, aspartyl β-hydroxylation, and proteolytic processing that removes the propeptide. The carboxylation status of a VKD protein can impact these subsequent post-translational modifications [38]. Subsequent secretion from the Golgi results in the VKD proteins localizing to several different sites. Most are secreted out of the cell, where they circulate in blood (e.g., hemostatic VKD proteins) or reside in the extracellular matrix (e.g., osteocalcin and MGP), while others are retained at the cell surface (i.e., the Transmembrane Gla Proteins).

**Figure 3:**
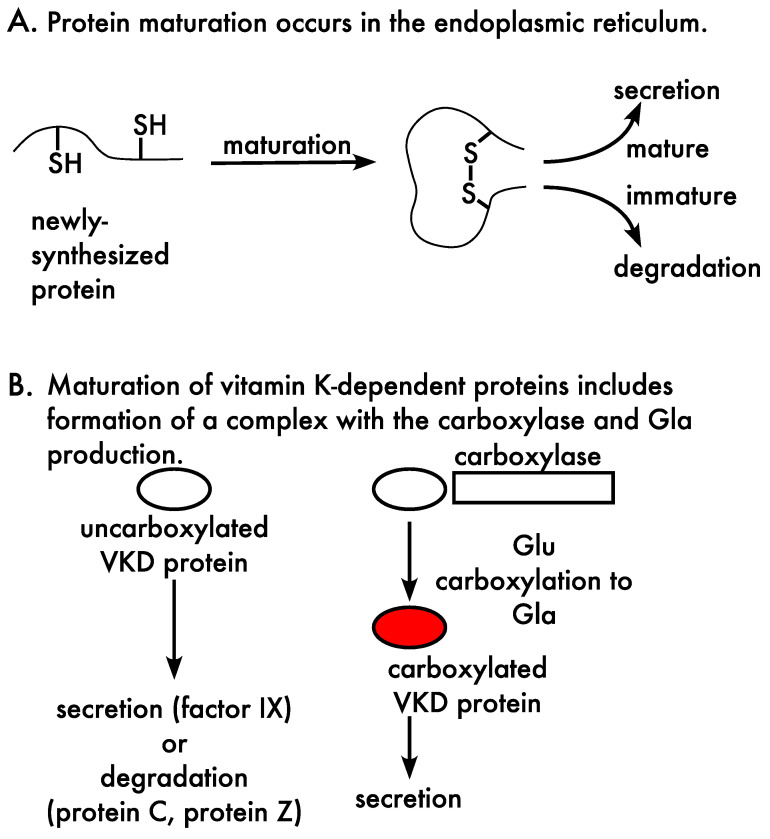
Quality control impacts the secretion of vitamin K-dependent proteins. (**A**) Newly-synthesized proteins undergo maturation that is mediated by chaperones and involves protein folding and, in some cases, disulfide bond formation and the assembly of complexes. Quality control mechanisms distinguish mature versus immature proteins, which are processed differently. Mature proteins exit the endoplasmic reticulum and traffic to the Golgi and beyond, while immature proteins are targeted for degradation through the ERAD pathway. (**B**) Vitamin K-dependent (VKD) protein maturation also includes Glu carboxylation to Gla that occurs in the endoplasmic reticulum. Cellular studies have shown that the fate of carboxylated and uncarboxylated proteins varies among individual VKD proteins. Specifically, both factor IX forms are secreted from cells, while only the carboxylated form is secreted in the case of protein C and protein Z.

The secretion of VKD proteins from the cell is impacted by quality control mechanisms in the endoplasmic reticulum, which ensure that only functional proteins and/or protein complexes are secreted (Figure 3A). Regulation by quality control involves chaperones that mediate protein maturation [39]. These proteins facilitate protein folding and disulfide bond formation, as well as assembly of multi-subunit complexes, which is relevant to VKD proteins because they form a complex with the carboxylase to undergo carboxylation. Regulation also includes ERAD (for endoplasmic reticulum associated degradation), which degrades proteins that are immature or that have not been properly assembled into complexes. A final regulatory step in quality control is the exit of proteins from the endoplasmic reticulum, where proteins are not taken up into vesicles for transport to the Golgi unless they are mature [40].

Protein maturation in the case of the VKD proteins includes an additional, unique step, i.e., Gla production (Figure 3B). The specific quality control components that impact the secretion of carboxylated VKD proteins have not been defined. However, cellular studies have shown that some VKD proteins (protein C, protein Z) exhibit preferential degradation of poorly carboxylated forms [41,42,43], while the secretion of a different VKD protein (factor IX) is unaffected by carboxylation status [44]. The secretion of proteins C and Z, then, involves a filtering process that results in the secreted proteins having a different carboxylation status from the intracellular products of carboxylation. Most VKD proteins have not been assessed for the impact of quality control. Secreted VKD proteins have been used as reporters of cellular carboxylation; however, the results from this indirect approach depend upon the impact of quality control. For example, analysis of VKD protein secreted from cells expressing a defective carboxylase mutant might reveal impaired carboxylation with factor IX as a reporter but not with protein Z, because filtering of uncarboxylated protein Z would mask the defect. Quality control mechanisms are also relevant to the expression levels of VKD proteins in studies analyzing secreted proteins. High level expression saturates the capacity for carboxylation, which would have different consequences for individual VKD proteins depending upon whether or not they are filtered by quality control mechanisms. The analysis of secreted proteins to study carboxylation therefore requires an understanding of how quality control mechanisms impact the secretion or degradation of un- or partially-carboxylated proteins.

### 3.2. The Carboxylation Reaction

VKD protein carboxylation is a complex process that involves several enzymes. The carboxylase converts multiple Glus to Glas in the Gla domains of VKD proteins, which generates a calcium-binding module that is essential for activity [2,45] (Figure 2). The carboxylase generates Gla residues by using the oxygenation of reduced vitamin K, i.e., vitamin K hydroquinone, which becomes converted to vitamin K epoxide during the reaction (Figure 2B). The vitamin K epoxide product is then recycled by a vitamin K oxidoreductase (VKOR) to regenerate the vitamin K hydroquinone, and this interconversion of reduced and oxidized vitamin K is referred to as the vitamin K cycle (Figure 2A). A single carboxylase modifies all VKD proteins, while two paralogs (VKORC1 and VKORC1L1) reduce vitamin K epoxide, and all three proteins are ubiquitously expressed [46]. VKORC1 and the carboxylase are essential for life, as mice lacking either gene die around birth from hemorrhaging [47,48]. Studies on mice lacking VKORC1 and/or the VKORC1L1 paralog indicate that VKORC1L1 supports VKD protein carboxylation during embryonic development, while VKORC1 is the main paralog important to hemostasis after birth [49].

Vitamin K refers to a family of forms that comprise a single phylloquinone and multiple menaquinones. These forms all have the same 2-methyl-1,4,-naphthoquinone nucleus, which is attached to a polyisoprenoid side chain. The side chain is more unsaturated in menaquinones, which vary in the number of isoprenoid units. High levels of both phylloquinone and menaquinone decrease vascular calcification in rodent models [50]. However, these vitamin K forms have differential bioavailability and tissue distribution that has made it challenging to understand the relative importance of these forms in human cardiovascular disease [51]. Their relative utilization as a cofactor for carboxylation is poorly understood, as analysis has only been performed on a limited number of vitamin K forms using a crude membrane preparation as the source of enzyme [52].

### 3.3. The Gamma-Glutamyl Carboxylase

The carboxylase is an integral membrane protein, which allows interaction with the hydrophobic vitamin K cofactor that is also membrane-embedded [53,54]. The carboxylase reaction is complex, using multiple substrates and cofactors. The carboxylase catalyzes a reaction between oxygen and reduced vitamin K to generate a vitamin K superbase that abstracts a hydrogen from Glu, generating a Glu carbanion that then reacts with CO_2_ to form Gla [2,45] (Figure 2B). The overall reaction is initiated by a Lysine (Lys218) that is regulated by substrate-assisted catalysis. Specifically, vitamin K epoxidation does not occur unless Glu residues are present [55]. This mechanism is important in preventing the vitamin K superbase from being formed and nonspecifically reacting with other molecules when Glu residues are not present.

Some, but not all, functional carboxylase regions have been identified. These regions include ones important to Glu binding, VKD protein interaction, catalysis and the Gla domain [55,56,57,58,59,60,61,62,63,64]. The carboxylase reaction is highly regulated; however, most of the residues that control the reaction remain to be determined. Allosteric interactions that regulate carboxylase activity include the stimulation of Glu catalysis when the EBD of VKD proteins bind to the carboxylase. In addition, the binding of both Glu and the EBD increases the affinity of the carboxylase for vitamin K. These allosteric interactions increase the efficiency of the reaction, and can also be important in the hemostatic response of patients with carboxylase mutations who are administered vitamin K [65,66]. A final and key regulatory mechanism is carboxylase processivity, which is essential for generating the multiple Gla residues that transform the Gla domain into a calcium-binding module (Figure 2C). Carboxylase processivity is the focus of Section 4.

### 3.4. Vitamin K Oxidoreductases (VKORs)

**Figure 4:**
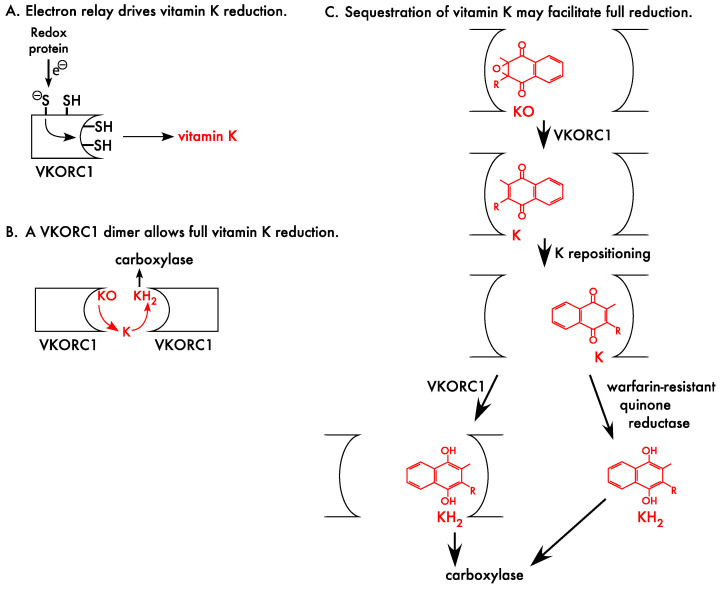
VKORC1 reduces vitamin K epoxide, and is inhibited by warfarin. (**A**) VKORC1 reduces vitamin K through an electron (e^−^) relay pathway. Redox protein generates thiols in VKORC1 located in the endoplasmic reticulum, which in turn generate membrane-embedded thiols that reduce vitamin K. (**B**) VKORC1 reduction of vitamin K epoxide (KO) to the vitamin K hydroquinone (KH_2_) carboxylase cofactor occurs in two reactions that involve a vitamin K quinone (K) intermediate. VKORC1 exists as a dimer [75], which can explain how VKORC1 performs both reactions. (**C**) Sequestration of the K intermediate may increase the efficiency of VKD protein carboxylation [75]. One monomer converts KO to the K intermediate, which is then repositioned for further reduction to KH_2_ by the second monomer. Warfarin uncouples the two VKORC1 reactions [76], which alters full reduction by inhibiting the K to KH_2_ reaction much more strongly that the KO to K reaction. Full reduction during warfarin therapy may therefore result from a second reductase cooperating with VKORC1 to generate the KH_2_ carboxylase cofactor.

The reduced vitamin K cofactor used by the carboxylase is generated by VKORC1 using Cysteine thiols [67,68,69,70]. VKORC1 is an integral membrane protein, like the carboxylase, and thiols in the lumen of the endoplasmic reticulum transfer electrons to membrane-embedded thiols that then reduce vitamin K (Figure 4A). VKORC1 becomes inactivated during vitamin K reduction, because the thiols are oxidized to disulfide bonds, and thiol regeneration is required for subsequent VKORC1 activity. VKORC1 activation occurs through a lumenal redox protein, and multiple candidate redox proteins have been suggested by thiol trapping studies (TMX1, TMX4 and ERp18) [71]. That study revealed disulfide linkage between VKORC1 and the candidates; however, these proteins also mediate quality control mechanisms, i.e., they are chaperones that mediate appropriate disulfide bond formation during protein maturation, which makes it difficult to assess whether these candidates have roles in quality control versus the electron relay pathway that activates VKORC1.

VKORC1 recycles vitamin K epoxide to the reduced form in two reactions, i.e., reduction of vitamin K epoxide to a quinone intermediate that is then reduced to vitamin K hydroquinone (Figure 4B). Each reaction requires one VKORC1 monomer, and the observation that VKORC1 exists as a dimer can explain how VKORC1 performs both reactions to accomplish full reduction of vitamin K epoxide to hydroquinone (Figure 4B). The two reactions are not equivalent, and only VKORC1 and the VKORC1L1 paralog perform the first reaction, i.e., vitamin K epoxide reduction. Quinone reductase activity other than VKORC1 that performs the second reaction, i.e., reducing vitamin K quinone to hydroquinone, has been observed [72,73,74]; however, the contribution to VKD protein carboxylation is poorly understood.

The VKORC1 dimer may facilitate the efficiency of full vitamin K epoxide reduction by sequestering the vitamin K quinone intermediate and effectively concentrating this form for reduction to vitamin K hydroquinone [75]. Vitamin K quinone intermediate generated by one monomer in the first reaction, then, would be reduced by the other monomer of the dimer (Figure 4C). This hypothesis is supported by studies with an inactive VKORC1 mutant that showed that the mutant is dominant negative in heterodimers with wild type VKORC1, decreasing vitamin K epoxide reduction and carboxylation [75]. Sequestration of vitamin K during reduction could be highly significant, as dietary and tissue vitamin K levels are low.

VKORC1 is the target of anticoagulants like warfarin (Coumadin), which is used to prevent blood clotting in patients, for example who have mechanical heart valves, deep vein thrombosis or pulmonary embolism. Warfarin uncouples normal VKORC1 reduction, which disrupts full vitamin K epoxide reduction by inhibiting quinone reduction much more strongly than epoxide reduction (Figure 4C). How warfarin uncouples VKORC1 full reduction of vitamin K epoxide is unknown, and one possibility is that warfarin binding interferes with the vitamin K movement between the two VKORC1 subunits that is required to accomplish full vitamin K epoxide reduction. Importantly, warfarin therapy dampens but does not abolish VKORC1 activity, which means that warfarin inhibited VKORC1 retains the ability to reduce vitamin K epoxide to the quinone form. This capability is significant because the low vitamin K levels in patients requires vitamin K recycling to support adequate VKD protein carboxylation. Recycling could occur with VKORC1 cooperating with a second reductase, i.e., a quinone reductase that reduces vitamin K quinone to hydroquinone, to drive VKD protein carboxylation (Figure 4C). The requirement for two reductases to accomplish full reduction would have significant consequences. Specifically, the quinone reductase may not be ubiquitously expressed like VKORC1, resulting in poor VKD protein carboxylation in those tissues lacking this reductase.

The response of patients to warfarin is variable, and some show bleeding complications during warfarin therapy that are rescued by vitamin K. Large amounts of vitamin K quinone are administered, which obviates the need for VKORC1 recycling because the high levels of vitamin K quinone can support VKD protein carboxylation through a quinone reductase. There have been significant efforts to identify the antidotal quinone reductase that rescues bleeding. Warfarin resistant quinone reductase activity has been demonstrated in liver [72,73,74], which is the major source of VKD clotting factors required for hemostasis. A long-standing hypothesis has been that the warfarin resistant quinone reductase is NQO1 (previously referred to as DT-diaphorase), which appears to have been ruled out by studies in *nqo1^−/−^* mice [77]. Another candidate is VKORC1L1, which has been shown to be warfarin resistant [78]. However, VKORC1L1 has been ruled out as the antidotal reductase by studies in mice [79], consistent with very low levels of this reductase in liver [49]. VKORC1 itself is a candidate antidotal reductase. While VKORC1 quinone reductase activity is poor during Warfarin therapy when vitamin K levels in tissue are low, because of warfarin uncoupling, VKORC1 could potentially facilitate hemostatic rescue when vitamin K levels are high. Recent studies have suggested that warfarin binding and inhibition of VKORC1 can be reversed by vitamin K [80,81]. However, other studies have concluded that warfarin inhibition is noncompetitive and irreversible [82,83]. Future studies that resolve the important question of how patients overdosed with warfarin are rescued by vitamin K will therefore be of significant interest.

## 4. Multiple Gla Residues Are Required for VKD Protein Function

VKD proteins contain multiple Gla residues, which form a highly organized network of contacts with several calcium ions [84]. The structural organization and functional consequence of the Gla domain has been extensively investigated in hemostatic proteins, which have a large number of Gla residues (i.e., 9–13). These studies show that the Gla domain inserts into the plasma membrane to facilitate protein-protein interactions important for the proteolytic reactions in the coagulation cascade. Osteocalcin, which only has three Gla residues, has been shown to have a very different structural organization in which each Gla residue only coordinates a single calcium in hydroxyapatite [85]. The functional consequence of this interaction is poorly understood. The structural organization of some VKD proteins remains to be determined, but would be highly informative in understanding their functions. MGP, for example, has been proposed to have two distinct roles, i.e., protein-protein interactions with bone morphogenic proteins [86] and binding calcium crystals [87]. The only structural study on MGP investigated MGP-derived peptides, and studies analogous to those performed on hemostatic VKD proteins could provide important insight into the function of MGP.

The presence of multiple Gla residues in VKD proteins raises the question of how the carboxylase accomplishes full carboxylation of the Gla domain. Other enzymes that act on substrates containing repetitive units have been shown to be processive [88], i.e., the enzyme remains bound to the substrate throughout multiple reactions. This processive property is essential to their various functions. A classic example of a processive enzyme is DNA polymerase, which remains associated with the DNA template during the multiple catalytic cycles of nucleotide addition in DNA replication. Processivity has been established in a large number of proteins that have diverse functions, e.g., ones that facilitate translation or transcription, molecular motors, nucleases, cellulases, ubiquitin ligases and kinases. We showed that the carboxylase is also a processive enzyme.

**Figure 5:**
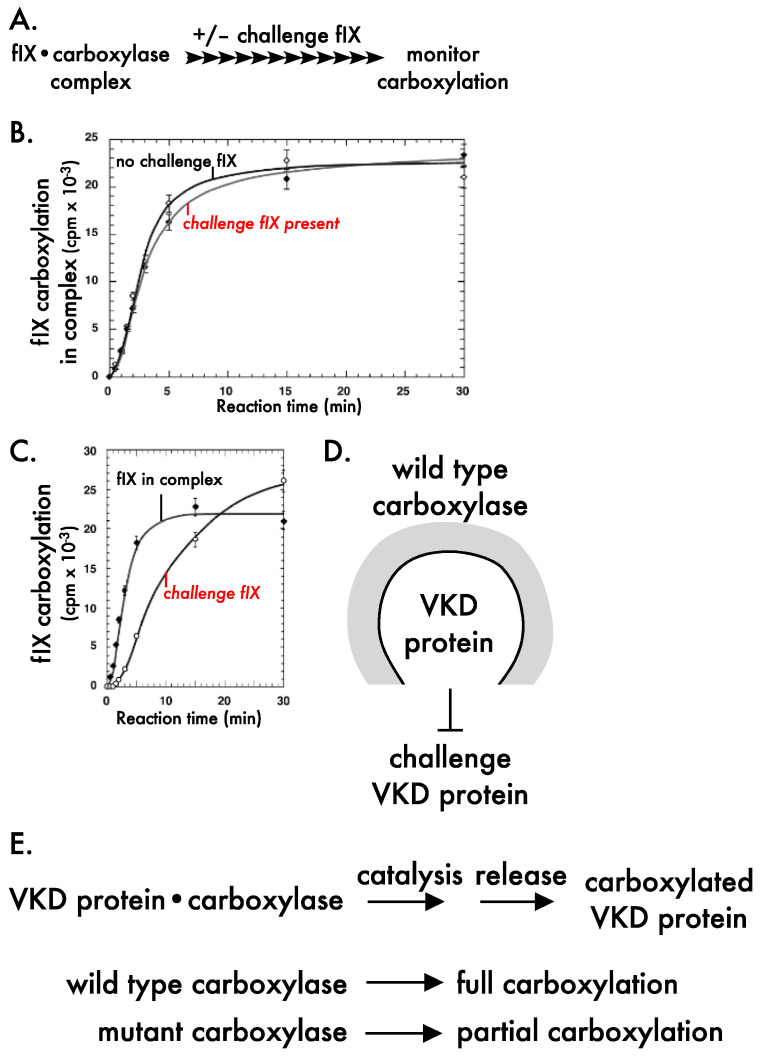
Carboxylase processivity regulates vitamin K-dependent carboxylation. (**A**) To study carboxylase processivity, a complex between the carboxylase and a vitamin K-dependent (VKD) protein is incubated in the presence or absence of a distinguishable VKD protein, and carboxylation of both VKD forms is monitored [16]. The VKD protein in the study was full length factor IX (fIX) attached to the propeptide that mediates complex formation. The challenge protein was the propeptide-containing fIX light chain that has the entire Gla domain. (**B**) Carboxylation of fIX in the complex is the same in the presence or absence of the challenge protein. (**C**) Carboxylation of the challenge protein occurs after carboxylation of fIX in the complex. (**D**) Wild type carboxylase blocks the access of challenge protein during the carboxylation of VKD protein in the complex [16]. (**E**) Carboxylase processivity depends upon the relative rates of catalysis versus release. The time to fully carboxylate the Gla domain is 5–6 fold faster than release with wild type carboxylase [15]. A mutant carboxylase with impaired processivity shows similar rates of catalysis and release and generates partially carboxylated VKD protein [15]. Panels B and C are adapted with permission from Stenina et al. [16], copyright 2001 American Chemical Society.

Carboxylase processivity was studied using a novel challenge assay that mimicked in vivo conditions. A VKD protein-carboxylase complex (i.e., factor IX-carboxylase) is incubated with a distinguishable VKD protein that is present in large excess over the complex (Figure 5A). In a processive mechanism, the VKD protein in the complex remains bound to the carboxylase throughout the multiple Glu conversions, and is unaffected by the challenge VKD protein. However, if the mechanism is nonprocessive, then VKD protein in the complex would be released and rebind the carboxylase during the multiple Glu modifications, resulting in competition with the challenge protein and a consequent decrease in carboxylation. Experiments to test these alternatives showed that carboxylation of factor IX in the complex was unaffected by the presence of the challenge factor IX (Figure 5B) and became fully carboxylated, and that the challenge factor IX was carboxylated after factor IX in the complex [16] (Figure 5C). Similar results have now been observed with a second VKD protein, i.e., MGP [15]. Two important conclusions from these studies are that VKD proteins are tethered to the carboxylase by the EBD throughout the multiple Glu to Gla carboxylation reactions, and that the carboxylase maintains a closed conformation that shields a VKD protein undergoing carboxylation, which allows the VKD protein to become fully carboxylated (Figure 5D).

Another important outcome of the studies came from rate analyses, which suggested that carboxylase processivity may not always result in the full carboxylation of VKD proteins. Full carboxylation of the Gla domain requires that the release of carboxylated VKD proteins is slower than the time to modify all Glas in the Gla domain, and we found that release is only 5–6 fold slower [15,16]. This observation is strikingly different from that of previous studies, which suggested that VKD protein release is 3000-fold slower than catalysis [89]. The difference in values is highly significant. A 3000-fold difference would indicate full carboxylation, while a 5–6 fold difference would suggest that wild type carboxylase sometimes generates partially carboxylated VKD proteins. The difference in values is almost certainly due to the types of VKD substrates analyzed. The 3000-fold value was obtained using small peptides derived from VKD proteins, i.e., peptides containing the EBD or a few Glu residues [89], while the 5–6 fold value was obtained using propeptide-containing, full-length VKD protein [15,16]. The natural VKD substrates have significant differences from the small peptides. For example, linkage between the EBD and Gla domain in the full-length VKD protein facilitates allosteric carboxylase mechanisms not recapitulated by small, unlinked peptides. In addition, only the full-length VKD protein is tethered to the carboxylase throughout multiple Glu modifications, which necessitates intramolecular movement in the Gla domain to position Glu residues for catalysis. The rate of Glu catalysis is slower as the number of Glu residues decreases, resulting in a nonlinear rate of carboxylation for a VKD protein-carboxylase complex (Figure 5B). This decreased rate could result in partially carboxylated VKD proteins if there are perturbations in the relative rates of catalysis and release. One such perturbation is mutations in the carboxylase, as described in the next section.

These studies raise new questions about VKD protein carboxylation, for example whether vitamin K nutriture or warfarin therapy impact processivity and consequent full carboxylation. Dietary vitamin K levels are low and vary significantly with the diet, and vitamin K levels control the rate of catalysis. Vitamin K deficiency may therefore impact processivity by altering the relative rates of catalysis versus release. In addition, vitamin K levels vary significantly in different tissues, raising the question of whether the efficiency of processivity is the same in all tissues. Warfarin therapy may also impact processivity, because warfarin inhibition of VKORC1 decreases the supply of reduced vitamin K and therefore lowers the rate of catalysis. Early studies on warfarin inhibition revealed heterogeneous forms of prothrombin [90,91], and impaired processivity could explain these observations. Another question is whether there are differences in the processive carboxylation of individual VKD proteins. The EBDs of VKD proteins show widely varying affinities for the carboxylase when analyzed biochemically [92], although those results have recently been questioned by the same laboratory based on cellular results [93]. Processive carboxylation of osteocalcin is of particular interest because it has an EBD affinity for the carboxylase that is orders of magnitude lower than the other VKD proteins. Osteocalcin has a high affinity site within the mature protein, which has not yet been identified but raises the question of whether two sites of VKD protein interaction with the carboxylase are important to processive carboxylation [1]. Finally, the studies raise questions about the mechanism of processive carboxylation. For example, while the closed carboxylase conformation allows a VKD protein to become carboxylated (Figure 5D), there must be a transition at the end of the reaction to allow the exit of the carboxylated VKD protein and entry of an uncarboxylated VKD protein. Studies have shown that this transition is not solely due to the VKD protein becoming carboxylated: VKD protein remains bound to the carboxylase long after full carboxylation in the absence of exogenous VKD protein, and release is accelerated by the presence of exogenous VKD protein [44]. Future studies that address these questions will be important for defining how carboxylase processivity impacts human health.

## 5. Disruption of Vitamin K-Dependent Protein Carboxylation in Disease

Impaired VKD protein carboxylation is associated with multiple pathologies that include bleeding, soft tissue calcification, chronic kidney disease, calciphylaxis, atherosclerosis, osteoarthritis and osteoporosis [94,95,96,97,98,99,100,101,102,103,104,105,106,107,108,109,110,111,112,113,114]. Nutritional deficiency of vitamin K impacts VKD protein carboxylation [115,116,117], as do mutations in the enzymes that facilitate carboxylation. Only one VKORC1 mutation causing impaired carboxylation has been identified, which causes severe bleeding [69] in VKCFD2 (vitamin K clotting factor deficiency) disease. It is worth noting that mutations in amino acids absolutely required for VKORC1 function, e.g., the four Cysteine residues that mediate vitamin K reduction, have not been identified in patients, consistent with VKORC1 being required for survival [47].

A large number of VKORC1 mutations cause warfarin resistance, which is the need for higher doses of warfarin to manage hemostasis [118]. The requirement for higher doses is enigmatic, as many of the mutations are in evolutionarily conserved residues that would suggest functional roles, and mutations typically decrease activity. A novel mechanism of warfarin resistance has been proposed in which VKORC1 mutants that are less active than wild type VKORC1 in the absence of warfarin become more active in its presence because wild type VKORC1 is more sensitive to warfarin inhibition [76]. Other studies propose that the mutants are more active than wild type VKORC1 even in the absence of warfarin [80]. However, many of these mutants were analyzed using an indirect cellular approach, and were inactive when tested in a separate study using a direct biochemical assay that monitored KO reduction [119]. Future studies on these VKORC1 mutants will be important in defining the mechanisms of warfarin resistance.

Carboxylase mutations also cause disease, which includes VKCFD1 that is associated with severe bleeding. Like VKORC1, mutations that are essential for carboxylase activity (e.g., Lys218 and His160 [55,64]) have not been identified in patients. Analyses that explain why the mutations cause VKCFD1 have been limited to only a small number of carboxylase mutants. These studies show that carboxylase mutations disrupt functional regions in the carboxylase, e.g., that are important to carboxylase interaction with Glus and the EBD in VKD proteins [57,58]. Some carboxylase mutations cause aberrant calcification in addition to a mild bleeding defect [9,11,13,120]. This phenotype is referred to as pseudoxanthoma elasticum (PXE)-like, because the calcification defect is similar to that observed in a second disease, PXE. Unlike PXE-like patients, coagulation is normal in PXE patients, who have mutations in a different gene, ABCC6, which encodes an efflux pump of an unknown metabolite [10,12]. PXE-like patients show excessive skin folding, and skin biopsies indicate abundant calcification, disruption of elastin fibers, and poor MGP carboxylation [9,11,13,120]. Most of the mutations causing PXE-like or VKCFD1 are in regions of the carboxylase of unknown function. These mutants have been tested for carboxylation in large scale cellular screens that revealed mutant differences in vitamin K responsiveness and in carboxylating different VKD proteins [121,122].

Regulatory regions of the carboxylase are largely unknown, and our studies revealed the importance of carboxylase processivity to human health. Combined heterozygosity of the carboxylase mutations Val255Met and Ser300Phe causes PXE-like, and mutant analysis using small peptides derived from VKD proteins did not explain the defect in the patient [9]. The approach with more natural VKD substrates was therefore adapted to study the mutants [15]. Val255Met was shown to be responsible for the survival of the patient, as Ser300Phe activity was extremely poor. Interestingly, when the Val255Met mutant was analyzed in the challenge assay (as in Figure 5A), the mutant showed strikingly different results from wild type carboxylase. Specifically, VKD protein in a carboxylase complex was carboxylated before the challenge protein with wild type carboxylase (similar to Figure 5C) but at the same time with the V255M mutant. This defect resulted in partially carboxylated VKD protein with substantially reduced activity. Similar results were obtained with factor IX that is important to hemostasis and MGP that regulates calcification, and the results explain defective clotting and aberrant calcification in the patient [15]. The studies are the first to show that carboxylase mutations can impair processivity, and they underscore the essentiality of carboxylase processivity in generating functional VKD proteins.

The studies raise new questions regarding the impact of impaired processivity on disease. One question is the functional consequence of defective processivity. The Val255Met mutant is responsible for the survival of the patient and yet generated partially carboxylated factor IX, indicating that full factor IX carboxylation is not absolutely required for function. Assessing the impact of impaired processivity on the activity of other VKD proteins should be informative. An interesting consequence of impaired processivity was the production of much higher levels of partially carboxylated factor IX and MGP by the Val255Met mutant than fully carboxylated VKD protein generated by wild type carboxylase [15]. Higher levels of partially carboxylated MGP are relevant to the phenotype of the proband’s mother and aunt, who are heterozygous for Val255Met and wild type carboxylases and show aberrant calcification in skin [9]. Their phenotype is puzzling, as carboxylase mutations are normally recessive. They are also heterozygous for the ABCC6 gene, i.e., harboring wild type ABCC6 and a mutant that is normally recessive, which led to a previous suggestion that heterozygosity in both the carboxylase and ABCC6 may explain the phenotype [9]. An alternative explanation is that their phenotype is due to higher levels of partially carboxylated MGP generated by Val255Met than fully carboxylated MGP produced by wild type carboxylase. An intriguing question is whether partially carboxylated MGP interferes with the function of fully carboxylated MGP because of similarities in structure. Thus, while uncarboxylated VKD proteins are thought to be totally unstructured, partially carboxylated forms may have some structure, consistent with the activity observed in partially carboxylated factor IX. Undercarboxylated and uncarboxylated VKD proteins, then, may have different physiological consequences in vivo.

## 6. Approaches for Analyzing VKD Protein Carboxylation

VKD protein carboxylation is commonly assessed using antibodies developed against large Gla- or Glu-containing peptides derived from individual VKD proteins. This approach was originally developed using antibody against the hemostatic VKD protein prothrombin, which is referred to as PIVKA (for protein induced by vitamin K absence). The PIVKA assay has been valuable, for example as a biomarker for poor prothrombin carboxylation in hepatocellular carcinoma [123,124]. Antibodies have also been developed for other hemostatic VKD proteins, as well as MGP and GRP [34,125], where the antibodies have been informative in defining their roles as inhibitors of calcification [33,50,125]. An anti-Gla antibody has also been developed that recognizes a consensus sequence for hemostatic VKD proteins [126], which can serve as a useful indicator of carboxylation [76,127].

Some considerations are important for interpreting results obtained using the antibody approach. One consideration is that the antibodies do not necessarily inform on the extent of carboxylation. The antibody recognizes a Gla-containing sequence, however even a few Glas could elicit antibody reactivity. The antibodies are sometimes referred to as conformation specific, however the number of Gla residues required to induce the conformational change is unknown. This point is underscored by a recent study that analyzed the same Val255Met mutant that we showed is impaired in processivity. The study concluded that the Val255Met mutant has wild type levels of carboxylation, using a conformation specific antibody that recognizes Gla but did not reveal partial carboxylation [122]. A second consideration is that the antibody approach is much more informative for studying disease when both carboxylated and uncarboxylated forms of a VKD protein are assessed, as well as VKD protein levels monitored with anti-VKD protein antibodies that recognize both carboxylated and uncarboxylated forms. These measurements are important because factors other than carboxylation may be altered in disease, e.g., changes in VKD protein expression or filtering by quality control mechanisms. A final consideration is that the antibody measurements are often made in blood; however, some proteins (e.g., MGP) are synthesized in a large number of tissues. Consequently, the carboxylation status of the mixture of circulating VKD proteins may not necessarily reflect that of VKD proteins in specific tissues.

Methods that have been developed to assess the extent of carboxylation and/or investigate individual Gla residues are Gla quantitation, Edman degradation and mass spectrometry. Gla quantitation involves hydrolyzing purified VKD proteins to amino acids, which are then separated and quantitated using HPLC [128]. This approach unequivocally determines the extent of carboxylation, and has been valuable in many studies, e.g., that assess the effect of VKD protein mutations on carboxylation or that define the carboxylation status of VKD protein expressed in cells [44,128,129,130,131]. A limitation of Gla quantitation is that it does not provide information about specific Gla residues. Edman degradation, which subjects purified proteins to cycles of peptide bond cleavage, is informative for analyzing specific Gla residues. This approach was used, for example, in defining the carboxylation status of MGP isolated from tissue [132]. A limitation with Edman degradation is that the specific Gla residues are a mixture of many VKD protein molecules, and this approach has now largely been replaced by mass spectrometry.

Mass spectrometry is a powerful approach to study carboxylation because it can identify each Glu/Gla residue in the Gla domain of an individual VKD protein molecule. One approach for studying VKD proteins is liquid chromatography mass spectrometry, in which peptide digests are fractionated on a column, and then ionized and bombarded to sequence the peptide. Bombardment results in the loss of the Gla side chain before cleavage of the peptide backbone, which precludes obtaining sequence information; however, modification of the Gla residues by methylation prevents Gla cleavage and allows sequencing of Gla-containing peptides [62]. This approach was valuable in identifying the Gla domain of the carboxylase [62]. VKD proteins have also been studied using MALDI-TOF mass spectrometry. Proteins/peptides are ionized, and the mass to charge ratio is then determined and compared to a library of mass spectra. This approach was quite informative in a study on circulating osteocalcin, which revealed a surprisingly heterogeneous population of intact and truncated osteocalcin forms [133]. Future studies that extend the mass spectrometry approaches for studying carboxylation should be of considerable interest.
